# Efficacy and Safety of Paracetamol and NSAIDs for Fever and Pain Management in Children with Chronic Diseases: A Narrative Review

**DOI:** 10.3390/children13010071

**Published:** 2026-01-01

**Authors:** Gregorio Paolo Milani, Giangiacomo Nicolini, Mara Cananzi, Luca Spiezia, Enrico Vidal

**Affiliations:** 1 Pediatric Unit, Foundation Istituto di Ricovero e Cura a Carattere Scientifico (IRCCS) Ca’ Granda Ospedale Maggiore Policlinico, 20122 Milan, Italy; gregorio.milani@unimi.it; 2 Department of Clinical Sciences and Community Health, University of Milan, 20122 Milan, Italy; 3 Pediatric Care Unit, Conegliano Hospital, 31015 Conegliano, Italy; giangiacomo.nicolini@aulss2.veneto.it; 4 Unit of Paediatric Gastroenterology, Digestive Endoscopy, Hepatology and Care of the Child with Liver Transplantation, Department of Women’s and Children’s Health, University Hospital of Padova, 35128 Padova, Italy; mara.cananzi@aopd.veneto.it; 5 General Medicine and Thrombotic and Haemorrhagic Diseases Unit, Department of Medicine, University of Padova, 35128 Padova, Italy; luca.spiezia@unipd.it; 6 Pediatric Nephrology Unit, University Hospital of Padova, 35128 Padova, Italy; 7 Department of Medicine (DMED), University of Udine, 33100 Udine, Italy; 8 Institute of Pediatric Research “Città Della Speranza”, 35127 Padova, Italy

**Keywords:** paracetamol, acetaminophen, NSAIDs, ibuprofen, pediatric, fever, pain, chronic disease

## Abstract

**Highlights:**

**What are the main findings?**

- Paracetamol and ibuprofen show comparable efficacy for fever and pain in children with chronic diseases, but differ in safety profiles.
- Caution is needed for the use of paracetamol in children with malnutrition, obesity and neuromuscular disorders, while NSAIDs carry higher risks of complications in patients with gastrointestinal, renal and coagulation diseases.

**What are the implications of the main findings?**

- Clinical decision making should integrate comorbidities, concomitant therapies, and infection status to optimize symptom control and minimize harm.
- Evidence is limited for many chronic pediatric conditions and new studies are warranted.

**Abstract:**

**Background/Objectives**: Fever and pain are among the most common symptoms in pediatric infections and chronic diseases, causing significant discomfort for children and concern for caregivers. Effective management is essential to relieve distress while avoiding overtreatment or undertreatment. Paracetamol and nonsteroidal anti-inflammatory drugs (NSAIDs), particularly ibuprofen, are the primary antipyretic and analgesic agents in pediatric care, but their use in children with chronic conditions might be challenging. **Methods**: A narrative review and clinical expert judgment were used to synthesize current evidence on the use of paracetamol and NSAIDs (especially ibuprofen) in children with some common chronic diseases. **Results:** Paracetamol is often considered a first-line option in several chronic conditions. Caution is warranted in children with pre-existing malnutrition, obesity, and neuromuscular disorders as these factors might increase the risk of hepatotoxicity. NSAIDs provide additional anti-inflammatory effects and comparable analgesic efficacy but should be used cautiously in some high-risk populations due to potential gastrointestinal, renal, and bleeding complications. Their use is contraindicated in children with dehydration, renal impairment, nephrotic syndrome relapses, while careful risk-benefit assessment is required in small and vulnerable neonates. Some data also suggests NSAIDs may worsen outcomes in certain acute bacterial and viral infections. Data on chronic infections such as tuberculosis, HIV, and viral hepatitis are limited, highlighting the need for further research. Combination therapy with paracetamol and ibuprofen may enhance analgesia in postoperative settings without significantly increasing adverse events. Overall, available evidence is limited and largely observational. **Conclusions**: This narrative review synthesizes current evidence and clinical expertise to provide practical guidance on the rational use of paracetamol and NSAIDs in children, emphasizing individualized therapy according to comorbidities, risk factors, and clinical context, particularly in vulnerable populations. A risk-adapted, evidence-based approach ensures optimal symptom control while minimizing harm, supporting safer, more effective, and family-centered care for children with fever and pain.

## 1. Introduction

Fever and pain are among the most common symptoms encountered in pediatric infections, representing primary reasons for medical consultation and significant sources of distress for both children and their caregivers [1]. Fever is a natural immune response to infection, while pain commonly results from inflammation or tissue injury associated with the infectious process.

Effective management of fever and pain in children remains a clinical challenge. “Fever phobia” often leads to over-treatment, driven by parents’ exaggerated concerns and misconceptions about the risks of severe conditions and potential complications of fever [2]. Conversely, pain in children is frequently under-recognized and inadequately treated, resulting in delayed and suboptimal analgesia and potential long-term negative consequences, such as altered pain perception [3].

Therefore, effective symptom management is essential not only to alleviate discomfort but also to prevent the risks associated with both over-treatment, such as unnecessary drug exposure, and under-treatment, such as persistent pain or distress. Addressing these issues requires a comprehensive and rational approach, including systematic assessment, targeted educational initiatives for caregivers and clinicians, and strict adherence to evidence-based guidelines to optimize the management of fever and pain in pediatric care [4].

Paracetamol (acetaminophen) and nonsteroidal anti-inflammatory drugs (NSAIDs), such as ibuprofen, are the most widely used antipyretic and analgesic agents in pediatric care. Paracetamol is generally considered the first-line therapy due to its favorable safety profile, particularly its gastrointestinal and renal tolerability, as well as its suitability for use from birth [5]. NSAIDs, on the other hand, provide additional anti-inflammatory effects and offer comparable efficacy for mild-to-moderate pain, but their use requires caution because of potential adverse effects [6].

Despite their widespread use, discrepancies persist in clinical practice regarding appropriate indications and the choice between paracetamol and NSAIDs, particularly in vulnerable pediatric populations.

This narrative review integrates current evidence with the authors’ clinical expertise to address the management of fever and pain in children. Particular emphasis is placed on the efficacy, safety, and clinical application of paracetamol and NSAIDs (especially ibuprofen), with special attention to vulnerable pediatric populations. The aim is not only to summarize the available literature but also to translate evidence and clinical experience into practical guidance that can support rational prescribing, optimize symptom control, and minimize risks in real-world pediatric practice.

## 2. Pathophysiology of Fever and Pain in Pediatric Infections

Fever represents a fundamental component of the host defense against infection, inflammation, or immune-mediated disorders, and should be regarded as an adaptive response rather than a disease in itself. When pathogens such as bacteria, viruses, fungi, or parasites invade the body, they are recognized by the innate immune system through pattern recognition receptors (PRRs), including Toll-like receptors (TLRs), expressed on macrophages and dendritic cells. These receptors detect conserved microbial motifs, known as pathogen-associated molecular patterns (PAMPs), such as lipopolysaccharides (LPS) of Gram-negative bacteria, and trigger the release of pro-inflammatory cytokines including interleukin-1 (IL-1), interleukin-6 (IL-6), and tumor necrosis factor-alpha (TNF-α). Acting as endogenous pyrogens, these cytokines reach the hypothalamus via the bloodstream and stimulate brain vascular endothelial cells to upregulate cyclooxygenase-2 (COX-2). This enzyme catalyzes the conversion of arachidonic acid into prostaglandin E2 (PGE2), the pivotal mediator of fever. By binding to EP3 receptors on thermoregulatory neurons in the median preoptic nucleus of the hypothalamus, PGE2 elevates the thermoregulatory set point, thereby activating heat-conserving and heat-generating mechanisms, such as vasoconstriction in the extremities, increased thermogenesis in brown adipose tissue through sympathetic norepinephrine release, and shivering induced via acetylcholine signaling [7]. This controlled rise in temperature promotes leukocyte activation, impairs microbial replication, and enhances antimicrobial defense, ultimately facilitating pathogen clearance. Once the infection is controlled, anti-inflammatory cytokines such as interleukin-10 (IL-10) and transforming growth factor-beta (TGF-β) counterbalance this process, restoring the hypothalamic set point and promoting heat dissipation via sweating and vasodilation [8].

Pain is another frequent and clinically relevant manifestation of pediatric infections. It results from the activation and sensitization of peripheral nociceptors—specialized sensory neurons that detect potentially harmful stimuli—by both microbial products and host-derived inflammatory mediators. Prostaglandins, bradykinin, and cytokines such as IL-1β and TNF-α lower the activation threshold of nociceptors, leading to increased pain sensitivity (hyperalgesia) [9]. Beyond signaling tissue injury and inflammation, pain contributes to significant functional impairment in children, including irritability, reduced oral intake, sleep disturbances, and limitations in daily activities. These consequences highlight the importance of careful evaluation and tailored management strategies, taking into account the child’s developmental stage and clinical context.

## 3. Pharmacological Properties of Paracetamol and NSAIDs

Paracetamol and NSAIDs share analgesic and antipyretic effects, yet they differ substantially in their mechanisms of action, pharmacokinetics, and safety profile.

NSAIDs act mainly by inhibiting the cyclooxygenase isoenzymes COX-1 and COX-2, thereby reducing the synthesis of prostaglandins that mediate inflammation, pain, and fever. This explains both their clinical efficacy—anti-inflammatory, analgesic, and antipyretic—and their adverse effects, including gastrointestinal irritation, platelet dysfunction, and renal toxicity, which result from prostaglandin depletion in protective pathways [10]. Paracetamol, in contrast, has negligible peripheral anti-inflammatory activity and acts predominantly at the central level. Its mechanism is not fully elucidated: inhibition of a cyclooxygenase variant in the brain and spinal cord (sometimes referred to as COX-3) has been proposed, although this remains controversial [11]. More consistently, its active metabolite N-arachidonoylphenolamine (AM404) appears to contribute to analgesia and antipyresis by enhancing endocannabinoid signaling and activating transient receptor potential channels (TRPV1 and TRPA1) [12].

Pharmacokinetic differences are also clinically relevant and strongly influenced by age and route of administration [13]. Orally administered paracetamol is rapidly absorbed from the gastrointestinal tract, reaching peak plasma concentrations within 30–60 min, and is metabolized mainly through glucuronidation and sulfation, with a minor fraction undergoing cytochrome P450-dependent oxidation to reactive intermediates. Its elimination half-life in children is about 2–3 h, requiring administration every 4–6 h. Ibuprofen and most other NSAIDs are also rapidly absorbed, with peak plasma levels reached within 1–2 h, and are metabolized primarily by hepatic oxidation and conjugation before renal excretion. The half-life of ibuprofen in children is approximately 1.8–2 h; however, its longer duration of clinical effect supports dosing every 6–8 h.

Importantly, the pharmacokinetics of paracetamol vary according to the route of administration, a consideration of particular relevance in neonates. Oral administration is generally reliable in older infants and children but may be unpredictable in neonates due to delayed gastric emptying and immature intestinal absorption. Rectal administration is frequently used in this age group; however, it is characterized by slow and highly variable absorption, lower and delayed peak plasma concentrations, and reduced bioavailability, often necessitating higher or loading doses to achieve therapeutic effects. In contrast, intravenous administration bypasses absorption variability, provides rapid and predictable systemic exposure, and is particularly advantageous in neonates and critically ill children when enteral administration is not feasible or reliable. Neonatal pharmacokinetics of paracetamol are further influenced by developmental immaturity of hepatic metabolic pathways, with sulfation predominating over glucuronidation and a prolonged elimination half-life compared with older children. These maturational differences underscore the need for route-specific and age-adjusted dosing strategies in the neonatal period [14].

Another relevant distinction concerns plasma protein binding: NSAIDs are highly bound to plasma proteins (typically >95%, predominantly albumin), a feature that significantly affects their distribution and pharmacodynamics [15]. In contrast, paracetamol exhibits low binding—around 15–25% under therapeutic conditions—which increases only slightly with higher albumin levels. Clinically, this has meaningful implications: hypoalbuminemia states, the free (active) fraction of NSAIDs may increase, potentially increasing toxicity, whereas paracetamol is less affected by changes in albumin levels due to its low protein binding [16].

These pharmacological differences underline the importance of careful drug selection in vulnerable pediatric populations: paracetamol is generally preferred for its favorable safety profile, whereas NSAIDs are valuable when anti-inflammatory activity is required, though at the cost of a narrower therapeutic margin.

In addition to developmental and clinical factors, interindividual variability in drug response may also be influenced by genetic determinants, a concept increasingly relevant to individualized therapy. Genetic polymorphisms affecting drug-metabolizing enzymes and molecular targets (e.g., CYP and UGT isoenzymes for paracetamol metabolism, and cyclooxygenase pathways for NSAIDs) have been proposed as potential modifiers of both pharmacokinetics and pharmacodynamics of analgesic and antipyretic agents [17].

However, for paracetamol and commonly used pediatric NSAIDs, current pharmacogenomic evidence remains limited, heterogeneous, and insufficient to support routine genotype-guided dosing in clinical practice. At present, age, weight, organ function, comorbidities, and clinical context remain the primary drivers of individualized therapy, while pharmacogenomics represents a promising but still exploratory component of personalized medicine in this field.

## 4. Safety Profile and Clinical Considerations of Paracetamol and Ibuprofen

### 4.1. Comparative Efficacy

Paracetamol and ibuprofen are both well-established first-line treatments for fever and mild-to-moderate pain in children. Their effectiveness in relieving pain and reducing fever-related discomfort has been widely demonstrated in numerous clinical trials [13,18,19,20,21,22]. Comparative studies and systematic reviews consistently show that their efficacy is broadly comparable for most pediatric indications, with no clear superiority of either drug in routine use [23,24,25,26]. Specifically, a 15 mg/kg dose of paracetamol is considered equivalent to a 10 mg/kg dose of ibuprofen in terms of therapeutic effect [27,28]. Although ibuprofen may provide a faster onset of action and a longer duration of fever reduction compared with paracetamol, these differences are generally neither statistically significant nor clinically decisive [25,26].

### 4.2. General Safety Overview

While their overall safety profiles are generally comparable, they differ in the types of adverse events reported [29,30]. At recommended doses, both drugs are associated with rare but specific side effects, making careful selection based on the clinical context essential. Therefore, the choice between paracetamol and ibuprofen should be individualized, balancing therapeutic benefits against potential risks, and guided by the child’s clinical status, underlying comorbidities, and specific risk factors for adverse effects, such as renal, hepatic, or gastrointestinal conditions. This tailored approach ensures safe and effective symptom management, particularly in vulnerable pediatric populations.

### 4.3. Specific Safety Considerations

#### 4.3.1. Gastrointestinal Effects

Prostaglandins play an important role in protecting the gastric mucosa from acid-related damage by inhibiting the production of hydrochloric acid by gastric parietal cells and by stimulating the production of protective mucus by the epithelial cells lining the stomach [31]. Treatment with NSAIDs disrupts this balance: on the one hand, suppression of prostaglandin synthesis removes the inhibitory control on parietal cell activation, leading to increased acid secretion; on the other, it reduces the protective mucus barrier that normally safeguard the mucosa, resulting in epithelial and endothelial injury as well as impaired local microcirculation. These pathophysiological alterations translate clinically into a spectrum of gastrointestinal manifestations, ranging from nonspecific dyspeptic symptoms to erosive gastritis, peptic ulcer disease, and, in severe cases, upper gastrointestinal bleeding or gastric perforation [32].

Beyond the stomach, NSAIDs can also exert deleterious effects on the small and large intestine. In this context, the pathogenic mechanisms differ from acid-mediated injury and involve enterohepatic recirculation of NSAID conjugates with bile acids. Once secreted into the intestinal lumen, these conjugates undergo bacterial deconjugation, releasing active drug metabolites that directly damage the epithelial surface and increase intestinal permeability [32]. The resulting loss of barrier integrity facilitates antigen and bacterial translocation, leading to immune activation and mucosal inflammation. In parallel, systemic inhibition of prostaglandin synthesis reduces mucosal blood flow and impairs reparative processes [31]. Collectively, these alterations manifest clinically as a range of intestinal complications, from occult bleeding and chronic anemia to intestinal ulceration [33].

A 2025 network meta-analysis (41 randomized trials including 4935 children with acute pain) [34] and a 2020 meta-analysis focusing on children under two years of age (7 studies including 27,932 children with acute pain or fever) [25] indicate that ibuprofen and paracetamol have comparable gastrointestinal safety profiles in pediatric populations. Across both analyses, there was no increased risk of serious events such as bleeding or ulceration, which were exceedingly rare, and mild, self-limiting symptoms—mainly abdominal discomfort or nausea—were reported only occasionally [25,34].

However, evidence from case-control studies and pharmacovigilance databases suggests that NSAIDs may carry a relatively higher risk of gastrointestinal bleeding compared with paracetamol. In two case-control studies conducted in Italy and France, including 486 and 177 children, respectively, recent NSAID exposure—predominantly ibuprofen—was significantly associated with upper gastrointestinal bleeding. Although the absolute incidence was very low, cases of bleeding were reported even after short treatment courses, with a median duration of four days [35,36]. These findings indicate that NSAID therapy warrants careful monitoring, particularly when higher doses are used or treatment is prolonged [33,35,37].

Children with chronic comorbidities are at particular risk of gastrointestinal complications from NSAID therapy. In children with chronic liver disease and portal hypertensive gastropathy, as well as in those with pre-existing gastric disorders such as *Helicobacter pylori* infection, peptic ulcer disease, or a history of gastrointestinal bleeding, NSAID use increases the likelihood of adverse outcomes, including mucosal ulceration or bleeding [32,33,37]. Similarly, in inflammatory bowel disease (IBD), NSAIDs may exacerbate disease activity or trigger flares of intestinal inflammation [32]. In these high-risk populations, paracetamol is generally considered the preferred first-line option for symptom control, whereas NSAIDs should be avoided or used with extreme caution under close monitoring [38].

In addition, concomitant drug exposures may further increase the gastrointestinal risk associated with NSAID use in children. Although few studies have specifically evaluated synergistic effects, the combined use of multiple agents—such as concurrent administration of more than one NSAID, platelet antiaggregants or anticoagulants, corticosteroids, or antibiotics—may heighten this risk and therefore warrants caution in clinical practice [32,33,37]. In pediatric rheumatology, although generally well tolerated, prolonged NSAID therapy, particularly when combined with corticosteroids, high-dose aspirin, or disease-modifying antirheumatic drugs, can increase the risk of gastric and duodenal ulcer formation [32,39,40]. In pediatric oncology, concurrent use of chemotherapeutic agents, corticosteroids, and treatment-related thrombocytopenia further increases susceptibility to gastrointestinal bleeding, and case series have reported hemorrhagic and ulcerative complications in this setting [33].

#### 4.3.2. Hepatological Effects

Most NSAIDs undergo extensive hepatic metabolism, primarily via cytochrome P450 isoenzymes. They are converted into inactive metabolites, which are then conjugated with glucuronic acid and excreted in the urine. Hepatic metabolism is generally efficient, and NSAIDs are usually well tolerated by the liver. Clinically significant ibuprofen-related hepatotoxicity is very rare and may occur either as a dose-dependent toxic injury, typically following overdose, or as an idiosyncratic reaction with immunoallergic features (fever, rash, eosinophilia, facial edema, and lymphadenopathy). When liver injury occurs, it is predominantly hepatocellular and most commonly characterized by asymptomatic elevations in serum aminotransferases; more rarely, it presents as acute cholestatic hepatitis and, exceptionally, as acute liver failure. In most instances, liver injury resolves after discontinuation of the drug [41,42,43].

Paracetamol is primarily metabolized in the liver through glucuronidation and sulfation, while a smaller proportion is oxidized by cytochrome P450 enzymes into the reactive metabolite N-acetyl-p-benzoquinone imine (NAPQI). Under physiological conditions, NAPQI is rapidly detoxified through conjugation with glutathione. However, when hepatic glutathione stores become depleted, as in cases of overdose, prolonged high-dose exposure or malnutrition, excessive NAPQI accumulation can cause hepatocellular injury, potentially leading to fatal acute liver failure [44]. In cases of suspected acute paracetamol overdose, determination of serum paracetamol concentration and application of the Rumack–Matthew nomogram are diagnostic. When the ingested dose and timing are uncertain, quantification of serum paracetamol–protein adducts (APAP-CYS), including 3-cysteine adducts, provides a sensitive and specific biomarker for acetaminophen-induced liver injury [45,46]. In children with chronic liver disease, it does not appear to increase the risk of hepatotoxicity when used within the recommended dosage range. Nevertheless, careful dose adjustment and strict avoidance of both intentional and unintentional overdose remain essential to prevent toxicity [47].

#### 4.3.3. Renal Effects

Glomerular blood flow is maintained by autoregulatory mechanisms (myogenic response and tubuloglomerular feedback) as well as by a balance of vasoactive mediators. Among these, prostaglandins—particularly PGE_2_ and PGI_2_—play a compensatory role by dilating the afferent arteriole and counteracting vasoconstrictors such as angiotensin II and catecholamines. This mechanism is especially important when the effective circulating volume is reduced. Cyclooxygenase inhibition by NSAIDs blunts prostaglandin-mediated vasodilation, thereby lowering renal blood flow and glomerular filtration rate (GFR), and predisposing to Acute Kidney Injury (AKI). Epidemiologically, NSAID-associated AKI accounts for a small but consistent proportion of pediatric AKI, around 2–3% in large cohorts [48,49,50], with most cases occurring in dehydrated children, even at recommended dosages.

Two main mechanisms underlie NSAID-induced AKI. The most common, accounting for the majority of cases, is hemodynamically mediated AKI caused by the loss of afferent arteriolar vasodilation. Less frequently, acute tubulointerstitial nephritis (ATIN) develops as an idiosyncratic, immune-mediated reaction [51].

The hemodynamic mechanism deserves particular emphasis: dehydration and volume depletion significantly increase susceptibility, while children with reduced nephron endowment such as those with congenital anomalies of the kidney and urinary tract (CAKUT) or preterm/“small vulnerable” neonates have limited autoregulatory reserve and are especially prone to NSAID-induced GFR decline [52,53]. In nephrotic relapses or other hypoalbuminemic states, the high albumin binding of many NSAIDs (>95%) implies a larger free (active) fraction when albumin levels are low, a pharmacokinetic shift that can amplify toxicity [54].

ATIN usually develops after several days to weeks of exposure and may be clinically subtle; recovery generally follows drug withdrawal, whereas renal biopsy is reserved for severe or atypical cases [55].

From a stewardship perspective, NSAIDs should be avoided in dehydrated children and used cautiously—or not at all—in those with CKD, CAKUT, small vulnerable neonates, or during nephrotic relapses (Table 1). In such contexts, alternative agents are generally preferred; if NSAIDs are considered, they should be administered only when euvolemia is ensured, at the lowest effective dose, and for the shortest duration, with careful monitoring of kidney function.

Another important consideration is polypharmacy: the concomitant use of ACE inhibitors/ARBs and/or diuretics creates an unfavorable combination of hemodynamic effects—sometimes referred to as a “triple whammy”—that further increases the risk of AKI [56]. In these contexts, careful patient selection and a preference for alternatives, such as paracetamol, are warranted.

#### 4.3.4. Infection-Related Concerns

The use of paracetamol and NSAIDs in pediatric patients has been increasingly studied due to the potential association of NSAIDs with an elevated risk of complications in acute bacterial and viral infections.

NSAIDs may worsen the clinical course of acute bacterial infections, particularly skin and soft tissue infections (SSTIs), as well as infections caused by *Streptococcus pyogenes* including invasive conditions such as sepsis, necrotizing fasciitis, pleuropneumonia, and meningitis [57,58,59,60].

The use of NSAIDs in certain acute viral infections, particularly SARS-CoV-2 and other respiratory virus, has been associated with poorer outcomes, including an increased risk of hospitalization, more severe complications (e.g., peritonsillar and retropharyngeal abscess, empyema and pulmonary cavitation), and bacterial superinfections during varicella infection [61,62,63,64,65,66,67,68,69,70,71,72,73,74,75,76,77,78,79,80,81,82,83,84,85,86,87].

The immunomodulatory effects of NSAIDs can mask the signs and symptoms of bacterial infection and impair host defense mechanisms, potentially reducing local antibacterial activity and increasing the risk of bacterial proliferation and related complications, particularly if NSAIDS are administered without concomitant antibiotic therapy [64,81].

Consequently, paracetamol is generally preferred for symptomatic management of several acute bacterial and viral infections, given its favorable safety profile and lack of association with increased infectious complications.

Conversely, there are few studies on the use of paracetamol and NSAIDs during chronic infections, such as tuberculosis (TB), HIV and hepatitis B and C, and none have been conducted in pediatric populations.

A study conducted in 2016 explored the potential of repurposing NSAIDs as adjunct therapies in tuberculosis treatment, highlighting both the anti-inflammatory and direct anti-mycobacterial properties of certain NSAIDs. NSAIDs can modulate inflammation, which is critical in TB, as the immune response contributes to tissue damage. Some NSAIDs, such as ibuprofen and aspirin, have also demonstrated direct activity against *Mycobacterium tuberculosis* in laboratory studies. However, potential risks include immunosuppression, delayed bacterial clearance, and reactivation of latent TB, highlighting the need for further research [88]. In 2017, Wu et al. evaluated whether NSAIDs use was associated with an increased risk of developing active tuberculosis in a large population. The study included more than 8000 NSAIDs users in Taiwan and found that they had a higher risk of developing active TB compared to non-users. Non-selective NSAIDs, such as ibuprofen and naproxen, were more strongly associated with TB risk than COX-2 selective inhibitors like celecoxib. The authors concluded that NSAID use is linked to an increased risk of incident active TB and recommended that clinicians exercise caution with NSAID use, especially in TB-endemic areas or in patients at risk of latent TB infection [89]. In 2023, Carranza et al. found that COX-2 inhibitors, particularly eterocoxib, reduced pro-inflammatory cytokines such as IL-1β, TNF-α, and IL-6 in infected blood, modulated genes associated with inflammation and immune regulation, and did not impair the immune system’s ability to control *M. tuberculosis* growth, suggesting that COX-2 inhibitors could be a promising adjunctive therapy in TB treatment [90]. However, a clinical trial conducted in 2024 in TB patients receiving standard therapy with or without etoricoxib demonstrated that etoricoxib reduced macrophage capacity to restrict *M. tuberculosis* growth, altered immune activation—including reduced pro-inflammatory cytokine responses—and impaired cellular immune control, despite normal bacterial clearance by antibiotics. These findings indicate that, although COX-2 inhibitors have anti-inflammatory benefits, etoricoxib may be harmful as an adjunct therapy in TB, potentially weakening key immune functions, especially macrophage-mediated bacterial control [91].

Regarding HIV infection, a 1994 study investigated the pharmacokinetic interaction between zidovudine (AZT), an antiretroviral used in HIV treatment, and chronic high-dose acetaminophen therapy in a patient. The study found that prolonged acetaminophen use appeared to induce glucuronidation, a metabolic pathway shared by both drugs. This enzyme induction likely accelerated the metabolism of zidovudine, potentially reducing its therapeutic concentration [92]. In 2009, a study investigated how NSAIDs influence the antiretroviral activity of nucleoside reverse transcriptase inhibitors (NRTIs) in HIV-1-infected T lymphocytes, focusing on the role of the multidrug resistance protein 4 (MRP4). The study found that NSAIDs enhanced the antiviral effect of several NRTIs by increasing their intracellular concentrations. This effect was attributed to inhibition of MRP4 activity rather than changes in drug metabolism or uptake, and the combination of NSAIDs and NRTIs led to greater suppression of HIV-1 replication. These findings suggested that NSAIDs can boost the effectiveness of NRTIs in HIV-1-infected T cells by inhibiting MRP4-mediated drug efflux, indicating a potential adjunctive role for NSAIDs in HIV therapy, although further clinical evaluation is required [93]. In the same year, Morelle et al. reported a case of a patient who developed acute kidney injury (AKI) and proximal tubule dysfunction due to a drug interaction between tenofovir, an antiretroviral used to treat HIV, and diclofenac. Tenofovir is nephrotoxic, particularly in the proximal tubules, but it usually causes problems only after prolonged exposure or in predisposed patients. Diclofenac, by reducing renal perfusion through prostaglandin inhibition, likely precipitated the toxicity. The authors concluded that caution is warranted when combining tenofovir with NSAIDs such as diclofenac [94].

Very few studies have examined the relationship between hepatitis and the use of paracetamol or NSAIDs. In 2020, a study evaluated the association between cyclooxygenase inhibitors—including NSAIDs and selective COX-2 inhibitors—and the risk of developing hepatocellular carcinoma (HCC) in patients with chronic hepatitis B infection. The results suggested that COX inhibitors may reduce chronic liver inflammation and fibrosis, both key drivers of carcinogenesis, thereby exerting a protective effect against HCC in this population. This study supports further investigation into COX inhibitors as adjunctive therapy to prevent liver cancer in chronic hepatitis B, with potential implications for future clinical guidelines [95]. However, in the same year, Lisotti et al. reported a case of a patient with chronic hepatitis B virus (HBV) infection who developed AKI and subsequently experienced upper gastrointestinal (GI) bleeding after NSAID use [96]. This case highlights the risks of NSAID use in patients with preexisting liver disease and kidney impairment. Caution is advised when prescribing or using NSAIDs in patients with multiple comorbidities affecting the liver and kidneys. Conversely, a recent study published in 2025 examined the impact of paracetamol use on the severity and clinical outcomes of patients with acute liver failure due to viral hepatitis. In a large cohort, patients with viral hepatitis who had taken acetaminophen showed more severe liver injury, including higher liver enzyme levels and worse coagulation parameters. Paracetamol use was associated with increased severity of acute liver failure, higher rates of complications such as hepatic encephalopathy and multi-organ failure, and worse clinical outcomes, including increased mortality and decreased likelihood of spontaneous recovery [97]. This study underscores the importance of careful pain and fever management in viral hepatitis, highlighting the potential risks of acetaminophen in individuals with already compromised liver function.

In conclusion, further studies are needed, particularly in the pediatric population, to evaluate the use of NSAIDs and paracetamol in chronic infections. At present, it is not possible to determine whether these drugs are beneficial or contraindicated.

#### 4.3.5. NSAIDs and Coagulation

NSAIDs inhibit prostaglandin endoperoxide H synthase (PGHS)—also known as cyclooxygenase (COX)—that converts arachidonic acid to prostaglandins, prostacyclin, and thromboxanes. In addition to provide analgesic, antipyretic, and anti-inflammatory effects, these drugs are key mediator of platelet aggregation due to a reduction in thromboxane A2 synthesis. This effect impairs platelet function and prolongs bleeding time, increasing the risk of bleeding complications [98,99].

In children, the bleeding risk associated with NSAIDs use is heightened in those with inherited or acquired bleeding disorders, such as immune thrombocytopenic purpura (ITP), Haemophilia A and B or von Willebrand disease (vWD), thrombocytopenia, and in perioperative settings like tonsillectomy [100,101].

In these high-risk groups, NSAIDs should be used with caution or avoided to prevent hemorrhagic events. Given these considerations, paracetamol is generally preferred in situations where bleeding risk is a concern, as it does not affect coagulation or platelet function.

Multimodal analgesia, a pharmacologic approach that combines several classes of medications, is widely recommended for the management of post-operative pain in children and adults [102]. In children, randomized controlled trials (RCTs) have shown that combination therapy with paracetamol and ibuprofen provides superior pain relief compared with paracetamol alone, especially at ibuprofen doses below 5 mg/kg [103,104]. The primary effect is dependent on both the dose and the dose ratio, and appears to both extend the duration of analgesia and increase its overall effectiveness [105].

Specifically, several studies in children undergoing tonsillectomy have shown that this fixed-dose combination provides safe and effective analgesia without significantly increasing the risk of post-operative tonsillar bleeding [106,107,108].

#### 4.3.6. Antypiretics and Asthma

The relationship between antipyretic use, specifically acetaminophen and ibuprofen, and wheezing and asthma exacerbations in children remains controversial. While asthma is a chronic inflammatory disease characterized by bronchial hyperreactivity and is often diagnosed after the age of five, wheezing is a clinical symptom that may reflect transient viral episodes or early signs of asthma, particularly in infants and toddlers. This distinction is critical, as the observed association with these medications may differ depending on the specific outcome assessed. A pivotal RCT by Phipatanakul et al. found no significant difference in asthma exacerbations between children with mild persistent asthma treated with as-needed acetaminophen versus ibuprofen [109]. Similarly, Lesko et al. reported a lower risk of asthma-related outpatient visits in ibuprofen users compared to acetaminophen users [110]; however, this was a post-hoc analysis of a large RCT and not specifically designed to assess asthma outcomes. Overall, these findings were supported by a meta-analysis, which suggested that ibuprofen was not associated with an increased risk of asthma risk and may even be protective in febrile children without pre-existing asthma, although it indicated a potential risk of exacerbation in children with pre-existing asthma [111].

More nuanced findings emerge when wheezing is used as the outcome. A cross-sectional ED-based study found that ibuprofen use was associated with a lower risk of wheezing in febrile children, whereas acetaminophen showed no significant association [112]. Importantly, the authors highlight that reduced ibuprofen use in wheezing children may reflect parental and clinical caution rather than a direct drug effect. A separate meta-analysis focusing on wheezing and asthma exacerbations also found no overall difference between the two medications [113]. Conversely, Sordillo et al. reported an association between infant antipyretic use and early childhood asthma, but this association was attenuated after adjusting for respiratory tract infections in early life [114].

Taken together, current evidence does not support a clear-cut causal relationship between ibuprofen use and an increased risk of asthma or wheezing. However, caution is warranted in specific subgroups, including children with known NSAID hypersensitivity, severe or uncontrolled asthma, or aspirin-exacerbated respiratory disease (AERD) [109,111].

#### 4.3.7. Neurodevelopmental and Neuromuscular Diseases

Although outside the primary scope of this review, concerns about prenatal paracetamol and neurodevelopmental disorders have been raised. Early observational studies suggested possible associations, but these were limited by methodological weaknesses. More robust evidence has clarified the issue: a large Swedish sibling control cohort [115] found no causal link with autism, attention deficit and hyperactivity disorders (ADHD), or intellectual disability, and a comprehensive umbrella review [116] confirmed that the overall weight of evidence does not support such an association.

These findings are reinforced by authoritative positions. The American College of Obstetricians and Gynecologists (ACOG) explicitly states in its 2025 Practice Advisory [117] that acetaminophen remains the first-line analgesic and antipyretic in pregnancy, with no evidence of a causal link to neurodevelopmental disorders. The American Academy of Pediatrics (AAP) [118] and the Society for Maternal-Fetal Medicine (SMFM) [119] share this stance, emphasizing that benefits outweigh theoretical risks.

Regulatory agencies have issued similar confirmations: the UK Medicines and Healthcare products Regulatory Agency (MHRA) [120], the European Medicines Agency together with AIFA [121], and the World Health Organization (WHO) [122] all conclude that current data do not justify changes in clinical practice.

Regarding children with neuromuscular diseases, while caution is always advised due to the heterogeneity of these conditions, there is no specific evidence suggesting that paracetamol should be routinary avoided in this population when used appropriately [123,124]. On the other hand, a few data have suggested that some patients with myopathies might be at higher risk of hepatotoxicity, including acute liver failure [125]. Recent studies have highlighted that paracetamol pharmacokinetics in children with spinal muscular atrophy compared to healthy controls might differ. This difference is likely due to different body weight and the disease itself, which could influence the volume of distribution [126]. Overall, caution in the use of paracetamol is advised in children with neuromuscular disorders also considering the heterogeneity of these conditions.

**Figure 1:**
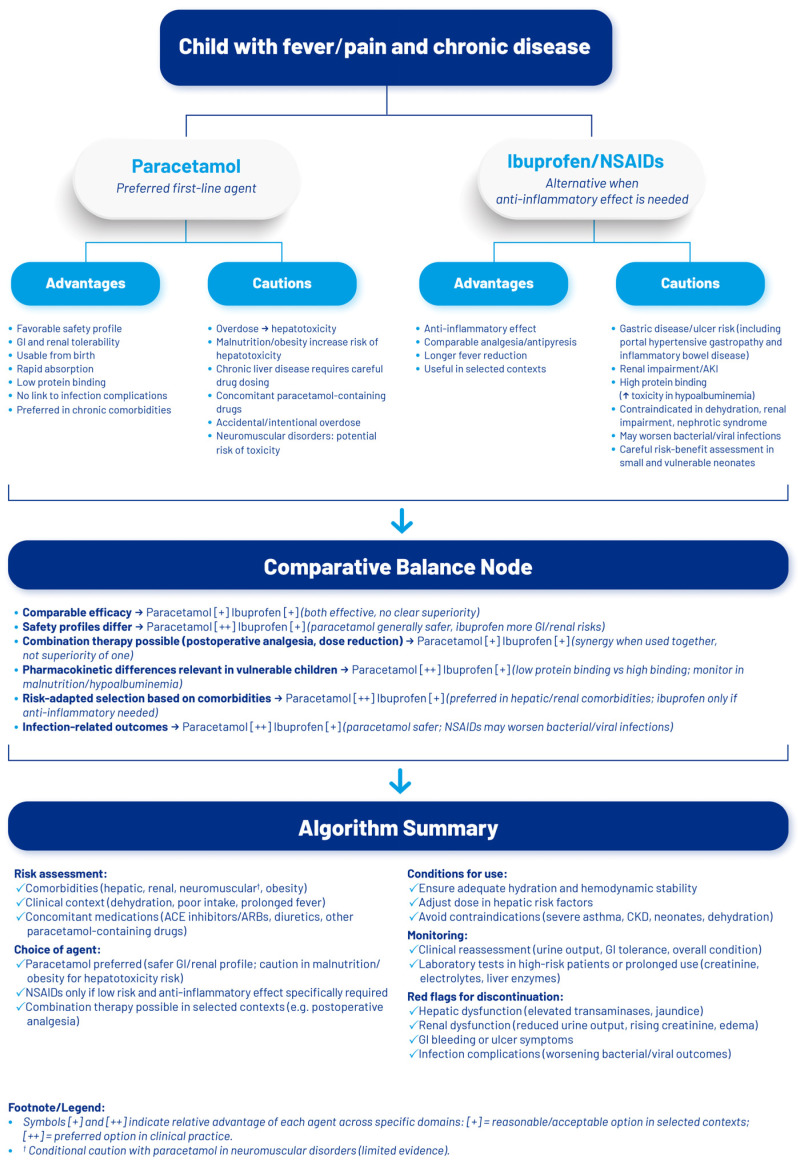
Risk-adapted decision algorithm for paracetamol and ibuprofen/NSAIDs use in children with chronic diseases.

To conclude the safety considerations, a comparative flowchart illustrating risk-adapted decision criteria for the use of paracetamol and NSAIDs in children with chronic diseases is presented in Figure 1.

## 5. Rational Use of Antipyretics and Analgesics in Vulnerable Pediatric Patients with Chronic Conditions

The primary purpose of antipyretic and analgesic therapy in children is to relieve discomfort rather than to normalize body temperature. International guidelines emphasize that fever per se is not an indication for treatment; pharmacological intervention should be considered only when the febrile state or associated pain results in significant distress, such as irritability, reduced oral intake, sleep disturbance, or impaired daily activity [27,28,127,128].

Paracetamol and ibuprofen are the only recommended agents for this purpose, and their selection should be guided by the child’s individual risk profile. Paracetamol is generally preferred as first-line therapy because of its favorable safety profile and minimal gastrointestinal, renal, or hematologic toxicity. Caution is required, however, in children with pre-existing liver disease, in which an appropriate dose is to be warranted, malnutrition, obesity [129], or concurrent use of other paracetamol-containing medicines, as these factors increase the risk of hepatotoxicity or overdose.

Ibuprofen can be particularly useful when anti-inflammatory effects are desired, such as in musculoskeletal pain or post-operative settings. Nevertheless, its use should be avoided in high-risk conditions, including dehydration, renal impairment, nephrotic syndrome relapses, and small vulnerable neonates. It should also be used with caution in children with gastrointestinal or coagulation disorders, and it should be avoided in those with severe asthma or known NSAID hypersensitivity.

The main advantages and contraindications of paracetamol and ibuprofen are summarized in Table 2, while Table 3 provides a practical overview of their use in children with complex conditions or comorbidities.

## 6. Combined or Alternating Use of Antipyretics

In the management of fever and pain in children, the alternating use of paracetamol and NSAIDs offers no clinical advantage over monotherapy and is therefore discouraged by current guidelines due to the lack of benefit and the increased risk of dosing errors and adverse effects [27,130,131,132,133].

Combined use of paracetamol and NSAIDs, particularly in low-dose fixed-dose formulations (FDCs), has a more favorable risk–benefit profile than alternating or full-dose combinations. For pain management, low-dose FDCs can provide a synergistic analgesic effect, with greater efficacy, longer duration, and faster onset of action without a significant increase in adverse events [134,135,136,137,138].

Current guidelines recommend reserving low-dose FDCs of paracetamol and ibuprofen for selected cases of persistent or moderate pain in children aged 2–12 years, when paracetamol or ibuprofen alone are not considered sufficient, such as acute pharyngotonsillitis, uncomplicated otitis media, or painful procedures like superficial abscess drainage [4,134,139,140]. In these settings, combination therapy may offer an optimal balance between efficacy and safety, while avoiding unnecessary dose escalation of individual agents.

On the other hand, the use of this combination in children with chronic conditions deserves further investigations.

## 7. Conclusions

Fever and pain are among the most frequent and challenging symptoms encountered in pediatric practice. Despite their prevalence, management remains often inconsistent, ranging from unnecessary overtreatment of fever to under-recognition and undertreatment of pain. A rational, evidence-based approach is essential to balance the relief of discomfort with the avoidance of iatrogenic harm.

Paracetamol and ibuprofen remain the cornerstones of symptomatic therapy, but their use must be tailored to the individual child. Paracetamol should be considered the first-line agent in several clinical situations, particularly in children with comorbidities that predispose to gastrointestinal, renal, or bleeding complications. Its use should be cautiously evaluated in children with malnutrition, obesity and neuromuscular disorders. Ibuprofen retains an important role when anti-inflammatory effects are desirable, but its use in children with chronic disease requires careful patient selection and vigilance. In the context of chronic infections, such as tuberculosis, HIV and chronic viral hepatitis, further studies in pediatric populations are needed to better define the benefits and risks of paracetamol and NSAIDs use. Overall, being a narrative review, these recommendations reflect synthesis of the limited available evidence and expert judgement and strength of these recommendations varies by domain.

Ultimately, optimal management requires pediatricians to adopt a risk-adapted and individualized strategy, grounded in systematic clinical assessment and in line with current evidence. This approach not only ensures effective relief of discomfort but also minimizes unnecessary drug exposure, prevents avoidable complications, and helps dispel persistent misconceptions about fever and its treatment. By integrating these principles into daily practice, clinicians can provide safer, more rational, and family-centered care for children with fever and pain.

## Figures and Tables

**Table 1:** Renal safety algorithm for antipyretic/analgesic use in children.

**1. Risk assessment**	▪ Clinical: dehydration, poor intake, prolonged fever. ▪ Renal: CKD, CAKUT, prematurity/“small vulnerable” neonates, history of AKI. ▪ Concomitant medications: ACE inhibitors/ARBs, diuretics, other nephrotoxins. ▪ Nutritional: hypoalbuminemia (e.g., nephrotic syndrome relapse).
**2. Choice of agent**	▪ In high-risk profiles, paracetamol is generally preferred given its limited impact on renal hemodynamics and low protein binding. ▪ NSAIDs may be considered when anti-inflammatory action is needed and risk is low.
**3. Conditions for NSAID use**	▪ Adequate hydration and hemodynamic stability. ▪ Avoidance of concomitant ACE inhibitors/ARBs and diuretics. ▪ Use of the lowest effective dose for the shortest duration.
**4. Monitoring**	▪ Clinical reassessment of urine output and overall condition. ▪ In higher-risk patients or with treatment >48–72 h, laboratory monitoring of creatinine and electrolytes may be appropriate.
**5. Red flags for discontinuation**	▪ Reduced urine output, rising creatinine, hyperkalemia, edema, or systemic features (rash, eosinophilia) suggestive of ATIN → prompt withdrawal of NSAID and reassessment.

**Table 2:** Advantages and contraindications of paracetamol and NSAIDs (ibuprofen) for the treatment of fever and pain in children.

	Advantages	Contraindications/Main Risks
**PARACETAMOL**	▪ Fewer gastrointestinal and renal side effects compared to NSAIDs ▪ Safe in children with gastrointestinal disorders, bleeding risk, or renal impairment ▪ Preferred in viral infections (e.g., varicella) ▪ No effect on platelet function or coagulation	▪ Hypersensitivity to paracetamol or excipients ▪ Concomitant use with other paracetamol-containing medicines ▪ Overdose risk, especially in malnutrition, obesity ▪ Case reports suggest a possible increased risk of toxicity in selected neuromuscular diseases (mainly myopathies)
**NSAIDs** **(Ibuprofen)**	▪ Rapid onset and longer duration of antipyretic action in some cases	▪ History of peptic ulcer disease, gastritis, Helicobacter Pylori infection, gastrointestinal bleeding ▪ History of inflammatory bowel disease ▪ History of portal hypertension and portal hypertensive gastropathy ▪ Concomitant use of other NSAIDs, corticosteroids or other drugs with potential gastrointestinal toxicity ▪ Renal impairment, dehydration, nephrotic syndrome, CAKUT, or prematurity (risk of AKI) ▪ Concomitant use with ACE inhibitors, diuretics, or other nephrotoxic drugs ▪ Varicella infection (risk of severe skin/soft tissue bacterial superinfection) ▪ Bacterial infections (notably Streptococcus pyogenes): potentially increased risk of invasive complications (e.g., sepsis, necrotizing fasciitis) even with early antibiotic use ▪ Bleeding disorders (ITP, vWD, thrombocytopenia), perioperative settings

**Table 3:** Use of paracetamol and ibuprofen in vulnerable pediatric patients with chronic conditions.

**Clinical Context**	**Preferred Option**	**Considerations/Contraindications**
**Small vulnerable neonates (prematurity, LBW, IUGR)**	Paracetamol	Avoid NSAIDs due to high nephrotoxic risk and limited safety data
**Chronic kidney disease, CAKUT, previous AKI**	Paracetamol	Avoid NSAIDs; monitor kidney function if unavoidable
**Nephrotic syndrome relapse/hypoalbuminemia**	Paracetamol	NSAIDs have higher free fraction, risk of nephrotoxicity
**Tuberculosis**	Not well defined	NSAIDs have conflicting studies about potential benefits (direct effect against Mycobacterium) and risks (immunosuppression and TB reactivation) No side effects of paracetamol
**HIV**	Not well defined	Interaction between either acetaminophen and NSAIDs and some antivirals Potential effect of some NSAIDs against HIV
**Gastrointestinal disease**	Paracetamol	Avoid NSAIDs or use with caution (risk for mucosal ulceration, bleeding and IBD flares)
**Coagulation disorders or perioperative setting**	Paracetamol	Avoid NSAIDs due to platelet inhibition and bleeding risk
**Asthma**	Either	Avoid NSAIDs in aspirin-exacerbated respiratory disease (AERD) or severe uncontrolled asthma
**Inflammatory pain**	Ibuprofen	Provides superior anti-inflammatory analgesia
**Neuromuscular disorder**	Not well defined	Caution advised in selected neuromuscular diseases (e.g., myopathies) with attention to dosing, repeated administration, and clinical monitoring

## Data Availability

No new data were created or analyzed in this study. Data sharing is not applicable to this article.
